# High Androgen Receptor mRNA Expression Is Associated with Improved Outcome in Patients with High-Risk Non-Muscle-Invasive Bladder Cancer

**DOI:** 10.3390/life11070642

**Published:** 2021-06-30

**Authors:** Danijel Sikic, Helge Taubert, Ralph M. Wirtz, Johannes Breyer, Markus Eckstein, Veronika Weyerer, Jennifer Kubon, Philipp Erben, Christian Bolenz, Maximilian Burger, Arndt Hartmann, Bernd Wullich, Sven Wach, Bastian Keck

**Affiliations:** 1 Department of Urology and Pediatric Urology, University Hospital Erlangen, Friedrich-Alexander Universität Erlangen-Nürnberg, 91054 Erlangen, Germany; helge.taubert@uk-erlangen.de (H.T.); jennifer.kubon@fau.de (J.K.); bernd.wullich@uk-erlangen.de (B.W.); sven.wach@uk-erlangen.de (S.W.), bastian.keck@web.de (B.K.); 2 Comprehensive Cancer Center Erlangen-EMN (CCC ER-EMN), 91054 Erlangen, Germany; markus.eckstein@uk-erlangen.de (M.E.); veronika.weyerer@uk-erlangen.de (V.W.); arndt.hartmann@uk-erlangen.de (A.H.); 3 STRATIFYER Molecular Pathology GmbH, 541713 Cologne, Germany; ralph.wirtz@STRATIFYER.de; 4 Caritas St. Josef Medical Center, Department of Urology, University of Regensburg, 93053 Regensburg, Germany; jbreyer@caritasstjosef.de (J.B.); mburger@caritasstjosef.de (M.B.); 5 Institute of Pathology, University Hospital Erlangen, Friedrich-Alexander Universität Erlangen-Nürnberg, 91054 Erlangen, Germany; 6 Department of Urology and Urosurgery, University Medical Centre Mannheim, 68167 Mannheim, Germany; philipp.erben@medma.uni-heidelberg.de; 7 Medical Faculty Mannheim, University of Heidelberg, 68167 Mannheim, Germany; 8 Department of Urology and Pediatric Urology, University Hospital Ulm, 89081 Ulm, Germany; christian.bolenz@uniklinik-ulm.de

**Keywords:** androgen receptor, bladder cancer, KRT5, KRT20, mRNA, NMIBC, PCR

## Abstract

The role of the androgen receptor (AR) in non-muscle-invasive bladder cancer (NMIBC) remains controversial. We retrospectively analyzed the mRNA expression of AR using RT-qPCR in 95 patients with high-risk NMIBC treated with a bladder-sparing approach and correlated AR with clinical data and recurrence-free survival (RFS), cancer-specific survival (CSS), and overall survival (OS). The mRNA expression of AR and KRT5, i.e., the basal-like subtype, was strongly correlated (rs = 0.456; *p* < 0.001). AR (*p* = 0.053) and KRT5 (*p* = 0.029) mRNA expression was negatively correlated with tumor grade. Kaplan–Meier analyses indicated significantly prolonged CSS (*p* = 0.020) and OS (*p* = 0.015) and a trend towards longer RFS (*p* = 0.051) in patients with high AR expression. High KRT5 expression was associated with significantly longer RFS (*p* = 0.033), CSS (*p* = 0.029) and OS (*p* = 0.030), while high KRT20 expression was associated with reduced RFS (*p* = 0.042). In multivariable analysis, none of the molecular markers was an independent prognostic factor. When performing a substratification with regard to molecular markers and clinicopathological parameters, high AR expression showed improved OS in patients with high KRT20 mRNA expression (*p* = 0.041). Women showed significantly longer OS in cases with high AR expression (*p* = 0.011). High AR was associated with significantly improved CSS in males (*p* = 0.044) and patients with instillation therapy (*p* = 0.040), while OS was improved regardless of instillation therapy. Younger patients with high AR expression had significantly improved RFS (*p* = 0.021), CSS (*p* = 0.014) and OS (*p* = 0.007). RFS was also improved in patients with high AR and low expression of either KRT5 (*p* = 0.003) or KRT20 (*p* = 0.014), but not in patients with high expression of KRT5 or KRT20. In conclusion, high AR mRNA expression is correlated with KRT5 mRNA expression and is associated with an improved outcome in high-risk NMIBC.

## 1. Introduction

Approximately 75% of newly diagnosed urothelial carcinomas of the bladder (UCB) are non-muscle-invasive bladder cancers (NMIBC) [1]. High-risk NMIBC is especially difficult to treat, given that recurrence and progression to muscle-invasive bladder cancer (MIBC) occurs in almost two-thirds of cases [2]. This can be overcome by early cystectomy with cancer-specific survival (CSS) in over 90% of cases; however, 40% of patients experience intra- and postoperative complications as well as the psychological burden of losing a functioning urinary bladder [3]. On the other hand, bladder-sparing strategies provide a chance for improved quality of life by keeping the patients’ own bladder. Nevertheless, 69% of patients receiving instillation therapy with Bacillus Calmette-Guérin (BCG) experience local or systemic side effects [4], while 35% of these patients have a recurrence of disease, and 31% have progression mostly within the first three years after diagnosis despite frequent cystoscopies and transurethral resections [5,6]. Currently, risk stratification and subsequent choice of treatment in NMIBC is based on the scoring system developed by the European Organization for Research and Treatment of Cancer (EORTC) in most cases [7]. Given that the EORTC scoring system is mainly based on clinicopathological features, molecular markers to aid and improve the present risk stratification systems are in high demand.

As with most cancers, UCB is more common in men [8]. The incidence of UCB is three to four times higher in men than in women, while women are diagnosed with more advanced tumors and have a worse course of disease after treatment [9]. These sex disparities have led to androgen receptor (AR) being one of the most prominent markers to be investigated as a potential diagnostic marker and therapeutic target in UCB [10]. Multiple preclinical experiments on cell lines and mice have suggested the involvement of AR and the androgen pathway in the development and progression of UCB [11,12,13,14,15]. In contrast, most retrospective analyses in patients with UCB revealed contradictory results regarding the role of AR in UCB [11,16,17,18]. We hypothesized that the use of immunohistochemistry as the main method of AR detection could be an important factor for these conflicting results. Therefore, we previously analyzed the prognostic role of AR mRNA expression in NMIBC and MIBC [19,20]. We found increased AR mRNA expression in NMIBC compared to MIBC [19]. While there was no prognostic relevance of AR mRNA expression in MIBC, in stage T1 NMIBC, we found that high AR mRNA expression was associated with improved survival and was an independent prognostic factor for improved recurrence-free survival (RFS) and CSS [20]. These promising results offer a rationale for the implementation of AR mRNA quantification in the diagnosis of high-risk NMIBC and new targeted therapies. However, these results still need validation. Therefore, the intention of the current study is to validate our previous results of the positive prognostic role of high AR mRNA expression within a new cohort of high-risk NMIBC using a reverse transcription quantitative real-time polymerase chain reaction (RT-qPCR)-based assessment. Moreover, we measured keratins KRT5 and KRT20 as surrogate markers for basal-like and luminal-like subtypes in UCB, respectively, analogous to our previous studies [20,21,22].

## 2. Materials and Methods

### 2.1. Patient Population

In this study, we retrospectively analyzed clinical and histopathological data from 95 patients treated with TUR-B at the Department of Urology and Pediatric Urology of the University Hospital Erlangen between 2000 and 2015 who were initially diagnosed with stage Ta or T1 NMIBC. The cohort is summarized in Table 1. All patients were treated with a bladder-preserving approach and received a second TUR-B within six to eight weeks after the initial TUR-B. An adjuvant instillation therapy with BCG or mitomycin was administered to 17 and 32 patients, respectively. According to standard treatment schemes, patients received at least six weekly BCG instillations or one mitomycin instillation per month. Tissue microarrays from formalin-fixed paraffin embedded (FFPE) tumor samples of all patients were evaluated for pathological stage according to the 2010 TNM classification and graded according to the common grading systems (WHO 1973, WHO 2016) by two experienced uropathologists (ME, AH). All specimens were categorized as high-risk NMIBC, meaning that either the tumor stage was T1 and/or grading was G3 according to the WHO 1973 classification. All specimens contained at least 50% tumor cells. Histopathological review confirmed that there were no histological variants present in the cohort. All procedures were performed in accordance with the ethical standards established in the 1964 Declaration of Helsinki and its later amendments. All patients treated after 2008 gave informed consent. For samples collected prior to 2008, the Ethics Committee in Erlangen waived the need for informed individual consent. The study was approved by the Ethics Committee of the University Hospital Erlangen (No. 3755 and No. 296_18 Bc).

### 2.2. Assessment of mRNA Expression by RT-qPCR

Tumor specimens were assessed by RT-qPCR as previously described [23]. In short, RNA was extracted from a single 10 μm curl of FFPE tissue and processed according to a commercially available bead-based extraction method (Xtract^®^ kit; STRATIFYER Molecular Pathology GmbH, Cologne, Germany). RNA was eluted with 100 μL of elution buffer and then stored at −80 °C until use.

The mRNA expression levels of the AR, KRT5 and KRT20 genes of interest as well as two reference genes, calmodulin-2 (CALM2) and β2-microglobulin (B2M), were determined by RT-qPCR, which involves the reverse transcription of RNA and subsequent amplification of cDNA executed in a one-step reaction. Each patient sample or control was analyzed in duplicate in an ABI Step One PCR System (Thermo Fisher Scientific, Waltham, MA, USA) according to the manufacturers’ instructions using the Invitrogen SuperScript III RT-qPCR system (Thermo Fisher Scientific) and gene-specific primer-probe combinations (STRATIFYER Molecular Pathology GmbH).

Forty amplification cycles were applied, and the cycle threshold (Ct) values for each of the three genes of interest were estimated as the mean of the duplicate measurements. These were then normalized against the mean expression levels of the reference genes (ΔCt method). The final values were generated by subtracting ΔCt from the total number of 40 cycles with a modification of the method by Schmittgen and Livak to ensure that normalized gene expression is proportional to the corresponding mRNA expression levels [24].

### 2.3. Statistical Methods

The correlations between the mRNA expression of AR, KRT5, and KRT20 and clinicopathological data were calculated using Spearman’s bivariate correlation or the chi2-test. Optimized cutoff values for each marker with regard to survival were defined by receiver operating characteristic (ROC) curves. For survival analysis, the time point zero was set as the date of the first TUR-B. The associations of mRNA expression with RFS, progression-free survival (PFS), CSS, and overall survival (OS) were determined by univariate (Kaplan–Meier analysis and Cox regression hazard models) and multivariate analyses (Cox regression hazard models, adjusted for age, sex, tumor stage, tumor grade according to the WHO 1973 classification, concomitant CIS, and instillation therapy). A *p*-value < 0.05 was considered statistically significant. The statistical analyses were performed with the SPSS 21.0 software package (SPSS Inc., Chicago, IL, USA).

## 3. Results

### 3.1. Correlation of the mRNA Expression of AR, KRT5, and KRT20 with Each Other and Clinicopathological Parameters

Nonparametric Spearman’s rank test displayed a significant positive correlation between the mRNA expression of AR and KRT5 (rs = 0.450; *p* < 0.001; Table 2). KRT20 was not significantly correlated with either AR or KRT5. Tumor grade was significantly negatively correlated with the expression of KRT5 (rs = −0.225; *p* = 0.029) and showed a trend for a negative correlation with AR (rs = −0.201; *p* = 0.053). KRT5 mRNA expression was negatively correlated with the overall survival status (alive: 0; dead: 1) (rs = −0.205; *p* = 0.046; Table 2).

### 3.2. Association of the mRNA Expression of AR, KRT5, and KRT20 with Survival

**Figure 1:**
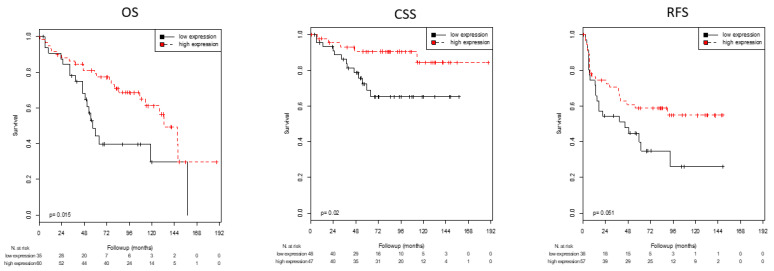
Kaplan–Meier analyses: Association of AR mRNA expression with OS, CSS and RFS. High AR mRNA expression was associated with improved OS (*p* = 0.015) and CSS (*p* = 0.020) and tended to be associated with better RFS (*p* = 0.051).

**Figure 2:**
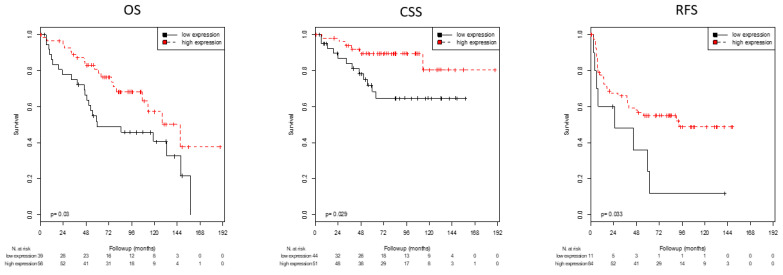
Kaplan–Meier analyses: Association of KRT5 mRNA expression with OS, CSS and RFS. High KRT5 mRNA expression was associated with improved OS (*p* = 0.030), CSS (*p* = 0.029) and RFS (*p* = 0.033).

**Figure 3:**
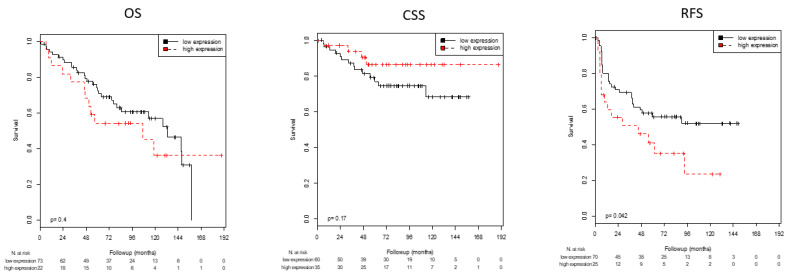
Kaplan–Meier analyses: Association of KRT20 mRNA expression with OS, CSS and RFS. High KRT20 mRNA expression was associated with shorter RFS (*p* = 0.042) but not with OS or CSS.

Optimal cutoff values for AR, KRT5 and KRT20 mRNA expression with regard to survival (RFS, PFS, CSS, and OS) were defined using ROC analyses (Table 3). In this way, high mRNA expression of AR was associated with significantly improved CSS (*p* = 0.020) and OS (0.015) and showed a strong trend towards longer RFS (*p* = 0.051; Table 3; Figure 1). High expression of the basal marker KRT5 was associated with significantly prolonged RFS (*p* = 0.033), CSS (*p* = 0.029), and OS (*p* = 0.030; Table 3; Figure 2). Patients with high expression of KRT20 demonstrated a statistically significant association with reduced RFS (*p* = 0.042; Table 3; Figure 3). There was no significant association between KRT20 expression and CSS or OS.

We did not consider PFS for further analysis because only eight patients had a documented time of progression, therefore rendering meaningful assessments difficult.

In multivariable analysis accounting for all markers—age, sex, tumor stage, tumor grade according to the WHO 1973 classification, concomitant CIS, and instillation therapy—none of the three molecular markers was an independent prognostic factor.

### 3.3. Association of AR, KRT5, and KRT20 with Survival Stratified by Clinicopathological Factors

The impact of hormones and hormone receptors is prone to age- and sex-dependent distinctions. Our current results also show different associations with molecular subtypes. Additionally, different outcomes of instillation therapies depending on AR expression have previously been discussed [25,26]. Therefore, we performed a substratification of the cohort to investigate the prognostic role of AR mRNA expression based on KRT5 and KRT20 expression, age, sex, and instillation therapy (Table 4).

#### 3.3.1. Association with KRT5 and KRT20

The median expression levels of KRT5 and KRT20 were used to distinguish patients with either high or low expression levels. In this way, stratification based on molecular subtypes showed significantly prolonged OS in patients with high AR mRNA expression in the group with high expression of KRT20 (*p* = 0.041; Table 4), while AR mRNA expression was not associated with OS or CSS for patients in the group with low KRT20 mRNA expression or the groups with any expression level of KRT5. High AR mRNA expression was also associated with significantly improved RFS in patients with low expression of KRT5 (*p* = 0.003) or KRT20 (*p* = 0.014; Table 4), but it was not associated with RFS for patients with high expression of KRT5 or KRT20.

#### 3.3.2. Association with Age

We used the median age of 71 years as a cutoff to define two age groups (≤71 vs. >71 years). In this way, patients in the younger age group with high AR mRNA expression showed a significantly longer RFS (*p* = 0.021), CSS (*p* = 0.014), and OS (*p* = 0.007; Table 4) than patients with low AR expression. In patients older than 71 years, no effect of AR mRNA expression on survival was observed.

#### 3.3.3. Association with Sex

When stratified according to sex, females with high AR mRNA expression had significantly improved OS (*p* = 0.011; Table 4), which was not the case for males. Interestingly, CSS was significantly prolonged in males with high AR mRNA expression (*p* = 0.044; Table 4) but not in females. There was no association between AR expression and RFS depending on sex.

#### 3.3.4. Association with Postoperative Instillation Therapy

Just over half of the patients (51.6%) received postoperative instillation therapy with mitomycin or BCG, which is comparable to a previous study [27].

Patients with high AR expression receiving instillation therapy had a significantly improved CSS (*p* = 0.043) and OS (*p* = 0.044; Table 4), whereas patients with high AR expression but without instillation had only a prolonged OS (*p* = 0.031; Table 4) but not CSS. AR expression was not associated with RFS with regard to receiving or not receiving instillation therapy.

## 4. Discussion

The role of AR in UCB has been the focus of many studies in recent years. Most preclinical analyses showed a direct involvement of the AR and androgen pathways in the development and progression of UCB, suggesting significant decreases in AR expression in UCB compared with benign urothelium and in high-grade/invasive tumors compared with low-grade/non-invasive tumors [14,28,29,30,31]. However, the prognostic role and therefore utilization in daily clinical routine remains highly controversial due to contradictory results in patients with UCB. Some studies described an association between AR positivity and tumor progression as well as worse outcome [11,16], while other studies reported high AR expression to be associated with reduced recurrence and an improved course of disease [17,18,32]. A recent meta-analysis reports a significant direct association between AR expression in NMIBC and RFS [33]. However, two earlier studies found no prognostic difference in patients with UCB based on AR expression [34,35]. Several factors might contribute to these inconsistent results. One reason might be the use of immunohistochemistry for AR detection at the protein level in UCB, which has been reported to vary between 12 and 53%, most likely because of different preservation techniques, staining protocols and scoring systems [36]. In contrast, AR at the mRNA level was previously shown to be detectable in all specimens of mouse models using RT-qPCR [12]. Another reason might be that many of the previously cited studies analyzed all stages of UCB together [16,18,34], although several studies suggested significantly higher AR expression in NMIBC than in MIBC [17,18,19,37,38], hinting at a different role of AR in NMIBC and MIBC. To overcome these weaknesses of analyzing AR in UCB, we recently investigated the prognostic role of AR in stage T1 NMIBC by quantifying AR mRNA using RT-qPCR. We were able to find a significant association of high AR expression with prolonged RFS, PFS, and CSS, with AR mRNA expression being an independent prognostic marker for improved RFS and PFS [20].

The goal of the present study was to reappraise the previous results within a new cohort of high-risk NMIBC patients. As before, we found a statistically significant association of high AR expression with prolonged CSS and a trend towards longer RFS in patients with high-risk NMIBC, thus confirming the positive prognostic effect of AR mRNA expression in high-risk NMIBC [20,32]. In addition, we found a significant association of high AR mRNA expression with OS. This was not found in our previous study, but this could be explained by the fact that the cohort in this study was younger (median 71 years compared to 75 years) compared to the previous study [20]. Although AR was not an independent prognostic marker for survival in the current cohort, a similar study by Yasui et al. found high AR mRNA expression to be an independent predictor for prolonged RFS by analyzing the role of AR mRNA expression in a cohort of 53 patients with either stage Ta or stage T1 NMIBC [39]. This was also the case in an immunohistochemistry-based study on 40 patients including all stages of NMIBC (pTis, pTa, and pT1) [40].

Additionally, in the present study, we were able to confirm the correlation of AR with KRT5, i.e., the basal-like subtype in NMIBC [20], which is unlike previous results in MIBC, showing an association of AR with the luminal-like subtype [41], supporting our theory that NMIBC and MIBC have a distinct pathophysiology necessitating separate analyses [19]. In addition to the positive correlation of AR with KRT5, we were also able to reiterate a favorable outcome in patients with a basal-like subtype [21]. In this study, patients with high KRT5 expression had significantly longer RFS, CSS and OS, while high KRT20 was associated with reduced RFS in high-risk NMIBC. Interestingly, when stratifying by molecular subtypes, patients exhibiting high AR mRNA expression in the high KRT20 expression group showed an improved OS compared to patients with low AR expression. Furthermore, patients with low expression of either KRT5 or KRT20 exhibited prolonged RFS when AR mRNA expression was high. These results imply an intertwining of molecular subtypes and AR expression as well as a prognostic benefit of high AR expression in patients with a luminal-like or non-basal subtype, who would otherwise have a worse outcome. Nevertheless, molecular subtypes are still a highly disputed topic, especially with regard to the markers necessary to define prognostically relevant subtypes [42,43,44]. Our results suggest that the addition of AR quantification to KRT5 and KRT20 might deliver additional prognostic information.

When stratifying our cohort by age, younger patients showed significantly better RFS, CSS, and OS in cases with high AR mRNA expression, while AR expression seemed to have no effect on survival in patients older than 71 years. These results are interesting but play into the general knowledge of the epidemiology and etiology of UCB. The risk of UCB is higher in the elderly population, as the average age at the time of diagnosis is 73 years [45]. At the same time, serum levels of hormones are known to decrease with increased age [46]. Our results imply a beneficial role of high AR expression, especially in young patients who usually exhibit higher serum androgen levels. Elderly patients might have no benefit of increased AR expression because of overall low androgen levels.

With regard to sex, our current results are interesting but appear inconsistent, as women with high AR expression have only significantly longer OS, while men with high AR expression have only prolonged CSS. The incidence and course of UCB disease are known to differ between the sexes. UCB is more frequent in males than in females, while females with UCB have a worse prognosis [9]. Our results support a protective role of androgens and AR on OS in women, who generally have lower androgen levels than men, while high AR expression might not have a prognostic impact on OS in men because of their overall high androgen levels. Finding an explanation for the positive effect of high AR on CSS in men and on OS in women is challenging, but because of the limited size of the sex groups, especially the female patients, future prospective studies are necessary to examine this result.

While we were able to confirm a better outcome in patients with high-risk NMIBC with and without instillation therapy when AR mRNA expression is high, the therapeutic implications remain unclear. Several retrospective studies in patients receiving androgen deprivation therapy for prostate cancer showed a reduced risk of developing UCB as well as reduced recurrence in patients with concomitant UCB [47,48]. What is even more interesting is that prostate cancer patients with NMIBC undergoing androgen deprivation therapy or therapy with 5α-reductase inhibitors showed a similar effect of a reduced risk of recurrence when high AR protein expression was observed [49,50]. Preclinical research showed that androgen deprivation or AR blockade augments the effect of intravesical instillation therapies such as doxorubicin and BCG by inducing oxidative stress and recruiting monocytes and macrophages that promote BCG attachment to UCB cells, respectively [25,26]. Therefore, patients with high-risk NMIBC receiving intravesical instillation therapy might benefit from an AR assessment.

However, AR also has a physiological role in the bladder and has been found to be present in the urothelium and bladder submucosa, such as smooth muscle cells and neurons [28,51]. Furthermore, AR is part of a DNA repair circuit and is involved in the response to genotoxic insults [52]. It is tempting to speculate that AR may strengthen the bladder wall and support physiological functions to prevent progression to MIBC.

There are several limitations to our study. First, this was a retrospective study with a relatively small cohort. Moreover, RT-qPCR for marker quantification is also associated with some inherent limitations, mainly that contamination with normal urothelium cannot be entirely excluded. We did not aim to replicate individual thresholds, although the interlaboratory variation for mRNA analysis appeared to be reasonably low [53]. We suggest that future prospective clinical trials may determine valid thresholds for better implementation of mRNA quantification in daily clinical practice. Altogether, we verified the association of AR and KRT5 mRNA expression with long-term prognostic outcomes in NMIBC patients.

## 5. Conclusions

In conclusion, we were able to confirm the positive prognostic role of high AR mRNA expression in high-risk NMIBC as well as the association with KRT5, i.e., the basal-like subtype, and a better prognosis, which might be helpful in the choice of further treatment. In addition, our results indicate a different role of AR in NMIBC after stratification by age or sex. Altogether, the role of AR, KRT5 and KRT20 in NMIBC, including stratification by sex, should be addressed further in future prospective studies.

## Figures and Tables

**Table 1:** Patient cohort.

Clinical Parameter	Variable	n (%)
Total cohort		95 (100)
Sex	Male	71 (74.7)
	Female	24 (25.3)
Median age years (IQR)	71 (64.5–79)	
Tumor stage	Ta	15 (15.8)
	T1	80 (84.2)
Tumor grade (WHO 1973)	G1	3 (3.1)
	G2	29 (30.6)
	G3	62 (65.3)
	NA	1 (1.0)
Concomitant CIS	Yes	31 (32.6)
	No/NA	64 (67.4)
Adjuvant instillation	Yes	49 (51.6)
	No	46 (48.4)
Median follow-up months (IQR)	69.0 (39.0–106.5)	
Recurrence-free survival (RFS)	Recurrence	50 (52.6)
	No recurrence	45 (47.4)
Progression-free survival (PFS)	Progression	8 (8.4)
	No progression	87 (91.6)
Cancer-specific survival (CSS)	Cancer-specific death	18 (18.9)
	Others	77 (81.1)
Overall survival (OS)	Dead	42 (44.2)
	Alive	53 (55.8)

IQR = interquartile range; NA = Not available.

**Table 2:** Spearman’s correlation test: Correlation of clinicopathological factors, prognostic factors and mRNA expression levels for AR, KRT5 and KRT20.

Parameter		Age	Sex	Grade	Stage	Instillation	OS	CSS	RFS	AR	KRT5	KRT20
Age	Correlation coefficient	1.000	0.113	0.095	0.060	0.020	0.200	0.192	0.121	−0.186	−0.096	0.117
	Sig. (2-sided)		0.277	0.365	0.562	0.850	0.052	0.062	0.244	0.072	0.354	0.259
Sex	Correlation coefficient		1.000	0.004	0.063	−0.030	0.068	0.090	−0.018	−0.004	−0.027	−0.042
	Sig. (2-sided)			0.967	0.545	0.772	0.514	0.387	0.863	0.966	0.799	0.683
Grading	Correlation coefficient			1.000	0.505	−0.282	0.075	0.111	0.033	−0.201	−0.225	0.044
	Sig. (2-sided)				**<0.001**	**0.006**	0.475	0.287	0.750	0.053	**0.029**	0.671
Stage	Correlation coefficient				1.000	−0.138	−0.054	−0.029	−0.205	−0.107	−0.113	−0.039
	Sig. (2-sided)					0.182	0.602	0.777	**0.046**	0.301	0.275	0.706
Instillation	Correlation coefficient					1.000	−0.099	0.123	0.220	−0.105	0.048	0.088
	Sig. (2-sided)						0.339	0.236	**0.032**	0.310	0.641	0.395
OS	Correlation coefficient						1.000	0.543	0.344	−0.083	−0.205	0.041
	Sig. (2-sided)							**<0.001**	**0.001**	0.426	**0.046**	0.693
CSS	Correlation coefficient							1.000	0.510	−0.133	−0.167	−0.120
	Sig. (2-sided)								**<0.001**	0.198	0.105	0.249
RFS	Correlation coefficient								1.000	−0.002	−0.039	0.096
	Sig. (2-sided)									0.988	0.706	0.354
AR	Correlation coefficient									1.000	0.450	0.001
	Sig. (2-sided)										**<0.001**	0.992
KRT5	Correlation coefficient										1.000	−0.052
	Sig. (2-sided)											0.618

Significant values are marked in bold. Sig. = Significance.

**Table 3:** Cutoff values for AR, KRT5, and KRT20 with regard to recurrence-free survival (RFS), progression-free survival (PFS), cancer-specific survival (CSS) and overall survival (OS) for patients within the NMIBC cohort (n = 95).

Marker	Survival	Cutoff Value (40-ΔCt)	High (n)	Low (n)	Survival in Months	*p*-Value
AR	RFS	32.9	57	38	93 vs. 62	(0.051)
	PFS	32.0	74	21	151 vs. 169	n.s.
	CSS	33.6	47	48	156 vs. 126	**0.020**
	OS	32.8	60	35	118 vs. 83	**0.015**
KRT5	RFS	32.1	84	11	85 vs. 43	**0.033**
	PFS	34.4	71	24	164 vs. 39	**0.037**
	CSS	36.4	51	44	153 vs. 125	**0.029**
	OS	36.0	56	39	120 vs. 86	**0.030**
KRT20	RFS	38.8	25	70	57 vs. 85	**0.042**
	PFS	37.9	45	50	169 vs. 149	**0.041**
	CSS	38.2	35	60	155 vs. 134	n.s.
	OS	38.9	22	73	99 vs. 107	n.s.

Significant values (*p* < 0.05) are marked in bold. Values *p* ≥ 0.05 to 0.1 are in parentheses. Values *p* > 0.1 are marked as not significant (n.s.).

**Table 4:** Association of AR mRNA expression and survival in NMIBC patients stratified by KRT5/KRT20 mRNA expression, sex, age, and instillation therapy.

Marker	Substratification Parameter	Survival	High (n)	Low (n)	Survival in Months	*p*-Value
AR	KRT5 low	RFS	20	18	112 vs. 49	**0.003**
	KRT5 high	RFS	37	10	71 vs. 89	n.s.
	KRT20 low	RFS	31	17	89 vs. 50	**0.014**
	KRT20 high	RFS	26	21	80 vs. 63	n.s.
	Age ≤ 71	RFS	31	17	108 vs. 58	**0.021**
	Age > 71	RFS	26	11	67 vs. 59	n.s.
	Male	RFS	43	28	90 vs. 61	n.s.
	Female	RFS	14	10	85 vs. 57	n.s.
	Instillation yes	RFS	24	22	78 vs. 52	n.s.
	Instillation no	RFS	33	16	91 vs. 66	n.s.
AR	KRT5 low	CSS	15	33	142 vs. 104	(0.057)
	KRT5 high	CSS	32	15	133 vs. 133	n.s.
	KRT20 low	CSS	24	24	140 vs. 108	(0.053)
	KRT20 high	CSS	23	24	155 vs. 132	n.s.
	Age ≤ 71	CSS	29	19	169 vs. 133	**0.014**
	Age > 71	CSS	18	29	112 vs. 102	n.s.
	Male	CSS	35	36	159 vs. 127	**0.044**
	Female	CSS	12	12	124 vs. 98	n.s.
	Instillation yes	CSS	19	27	158 vs. 115	**0.043**
	Instillation no	CSS	28	21	134 vs. 121	n.s.
AR	KRT5 low	OS	23	25	105 vs. 76	n.s.
	KRT5 high	OS	37	10	113 vs. 98	n.s.
	KRT20 low	OS	33	15	117 vs. 89	n.s.
	KRT20 high	OS	27	20	118 vs. 81	**0.041**
	Age ≤71	OS	34	14	140 vs. 82	**0.007**
	Age > 71	OS	26	21	87 vs. 76	n.s.
	Male	OS	45	26	116 vs. 88	n.s.
	Female	OS	15	9	113 vs. 64	**0.011**
	Instillation yes	OS	27	19	134 vs. 88	**0.044**
	Instillation no	OS	33	16	101 vs. 73	**0.031**

Significant values (*p* < 0.05) are marked in bold. Values *p* ≥ 0.05 to 0.1 are in parentheses. Values *p* > 0.1 are marked as not significant (n.s.).

## Data Availability

The data presented in this study are available on request from the corresponding author.
